# Supplementary material for: Prevalence and intensity of soil-transmitted helminths infection among individuals in model and non-model households, South West Ethiopia: A comparative cross-sectional community based study
Source: PLoS One. 2022 Oct 17;17(10):e0276137. doi: 10.1371/journal.pone.0276137 (PMC9576057; doi:10.1371/journal.pone.0276137)
Supplement: Attachment — Submitted filename: PONE-D-21-35319.docx [file pone.0276137.s001.docx]

**EDITOR’S COMMENTS**

Dear Authors, kindly respond adequately to the points raised by the reviewers. There is need for improvement in the language structure. Clear explanations should be given on the selection of study participants, and study design used.

**COMMENTS TO AUTHORS**

**FIRST REVIEWER:**

First of all, I would appreciate and thanks authors for their interest and work to address evidence gaps on neglected tropical diseases.
**General comments:**
--I would expect specific target groups in the title and I suggest the title to be shaped as “Prevalence and Intensity of Soil-Transmitted Helminths Infection among individuals in Model and Non-Model Households, South West Ethiopia: A Comparative Cross Sectional Community Based Study”
-There is inconsistent use of the word STH and STHs in the manuscript.
- Given the main focus of your study is on STHs, I was wondering about the relevance of including other intestinal parasites in this study since
-How did authors categorize households as model and non-model?
-Lines 153-154, how was the wet mount examination performed (how specimens collected, transported, and where performed?).
-The sampling technique need to be clearly stated. Explanation for inclusion criteria of study participants should be stated y in the study?
-I suggest the word education (literate/illiterate) in your manuscript to be categorized by using other references? From my point of view, I don’t recommend using the word ‘illiterate’.
-The age category is not consistent in the manuscript; please revisit in the results section.
- The entire manuscript should be checked and proofread; there are language uses, typographical errors, and grammatical lapses that can be very distracting.
-To strengthen the paper more, the authors can suggest concrete recommendations on how the gaps in the program can be addressed.
-Authors suggestion concerning others operational research would be very important.

**Specific comments:**
The abstract should:
-Before stating the evidence gaps exist on the effect of modeling households on prevalence of soil-transmitted helminths among individuals, abstract should high light brief information about soil-transmitted helminths for readers outside the filed?
-Authors should mention the place where the study was done.
Methods:
- It seems that the authors aimed at testing the hypothesis of ‘individuals in non-model would have a higher prevalence of sol-transmitted helminths than individuals in the model households’. And by taking this into consideration, I was wondering how a cross-sectional study can testify this concept? For such kinds of study, I thought cohort study design would a good option to assess the effect of exposure on the outcome.
-How study participants were selected? Further explanation should be stated on how participants are selected to get a representative study population from model and non-model households? Did author consider stratification in the sampling technique.
--The inclusion criteria should be stated in the study population section.
-Did you include children under 15 years old in the study? State if assent was obtained from children in the ethical statement.
-The laboratory technique should be mentioned and explained as part of the data collection procedure.
- Please describe all the variables that you collected data on in the methods section.
-The data analysis procedure was not well stated and presented. The multivariate logistic regression model considered model and non-model households as one variable; by taking this into consideration how would author made a comparative analysis between those groups to draw a conclusion based on the data presented in the study.
-Please mention the ethical approval number if the study was reviewed and approved.
Results, discussion, and conclusions:
-Explain how the results related to the hypothesis presented as the basis of the study.
- The discussion provides a concise explanation of the implications of the findings, particularly in relation to previous related studies and potential future directions for research. Results should not be restated in the discussion section.
-The conclusion should be drawn based on the data presented in the study.
References:
-Please check all references according to the journals’ guidelines.

**SECOND REVIEWER:**

The title is very interesting and the manuscript technically sound, and the data support the conclusions. The analysis sounds to have significance on the prevention and control of STHs infection.  The data underlying the findings in the manuscript fully available. The language that the authors used to write this manuscript is good. So with minor correction it can be considered for publication
