# Supplementary material for: Prevalence and intensity of soil-transmitted helminths infection among individuals in model and non-model households, South West Ethiopia: A comparative cross-sectional community based study
Source: PLoS One. 2022 Oct 17;17(10):e0276137. doi: 10.1371/journal.pone.0276137 (PMC9576057; doi:10.1371/journal.pone.0276137)
Supplement: Attachment — Submitted filename: Response to reviewers.docx [file pone.0276137.s003.docx]

Response to the Reviewers and editor:

We would like to extend our gratitude to the editor and reviewers in charge of evaluating our manuscript for fitting the journals publishing requirements.

- We have checked the references list; however, we have no article retracted from the journals.
- Thank you the third reviewer for finding our manuscript interesting and could have merit to the field, we appreciate.
- We accept the critical and important comment raised on the mean calculation. However, we prefer to use the arithmetic mean calculation for the sake of simplicity and we have made corrections accordingly such as including zero intensity counts in negative individuals to calculate the mean and updating in the text.
- We made also similar corrections in the main text indicating the way data was analyzed for the methods section.
- We have added the limitation of our study under the discussion section separately.
