# Supplementary material for: Prevalence and intensity of soil-transmitted helminths infection among individuals in model and non-model households, South West Ethiopia: A comparative cross-sectional community based study
Source: PLoS One. 2022 Oct 17;17(10):e0276137. doi: 10.1371/journal.pone.0276137 (PMC9576057; doi:10.1371/journal.pone.0276137)
Supplement: Attachment — Submitted filename: Alemu_PONE-D-21-35319R2_Comments.docx [file pone.0276137.s004.docx]

**PONE-D-21-35319R2: Reviewer’s comments**

**Summary**

The author have revised the manuscript (discussion) and highlighted the lack of baseline data as a major limitation. Thus, the manuscript may be considered for publication. There are a few minor comments below.

**Specific comments**

1. It is important to make it clear what the abbreviation HH is referring to because it appears to be interchangeably used to refer to, 1) household heads, and/or 2) households.
2. Line 24-27: Check grammar for the statement reading “This study…(HEP)”
3. Line 37: Insert the word “to be” between the words found and 32.4%.
