# Supplementary material for: Prevalence and intensity of soil-transmitted helminths infection among individuals in model and non-model households, South West Ethiopia: A comparative cross-sectional community based study
Source: PLoS One. 2022 Oct 17;17(10):e0276137. doi: 10.1371/journal.pone.0276137 (PMC9576057; doi:10.1371/journal.pone.0276137)
Supplement: Attachment — Submitted filename: Response to Reviewers.docx [file pone.0276137.s005.docx]

**PONE-D-21-35319R2: Reviewer’s comments**

(We would like to extend our appreciation to the editor handling our manuscripts review process once again)

**Summary**

The author have revised the manuscript (discussion) and highlighted the lack of baseline data as a major limitation. Thus, the manuscript may be considered for publication. There are a few minor comments below.

(We are grateful to the reviewers for their critical comments and corrective suggestions in strengthening our manuscript to be considered for publication. We have responded to the recent comments accordingly in this version.

In the previous version, we have tried to respond forwarding our revised version on raised comments related with data analysis and limitation of our study. To remind, we have incorporated the limitation of our study after the conclusion section of the main text in our former communication.

Whereas, it seems that the issue related with the mean calculation remains unsatisfactory. As a result, we have revised our current version incorporating such comments as well. For the calculation of geometric mean, we have included zero counts in the mean calculation after adding one to each datasets and subtracting one from the result of converted logarithmic calculations)

**Specific comments**

1. It is important to make it clear what the abbreviation HH is referring to because it appears to be interchangeably used to refer to, 1) household heads, and/or 2) households.

(Thank you for the comment, we have rewritten based on their concept as HH for household, HHs for household heads and also identified separately in the text whenever it refers to the individual members in the HHs accordingly)

2. Line 24-27: Check grammar for the statement reading “This study…(HEP)”

(Thank you, We made adjustment to the text in response to your suggested comment)

3. Line 37: Insert the word “to be” between the words found and 32.4%.

(Noted and we have incorporated the comment in the text)
